# Global Disparities in Premature Mortality

**DOI:** 10.1001/jamahealthforum.2025.3479

**Published:** 2025-10-03

**Authors:** Omar Karlsson, Dean T. Jamison, Gavin Yamey, Sarah Bolongaita, Wenhui Mao, Angela Y. Chang, Ole F. Norheim, Osondu Ogbuoji, Stéphane Verguet

**Affiliations:** 1 Duke University Population Research Institute, Duke University, Durham, North Carolina; 2 Centre for Economic Demography, School of Economics and Management, Lund University, Lund, Sweden; 3 Department of Epidemiology and Biostatistics, University of California, San Francisco; 4 Institute for Global Health Sciences, University of California, San Francisco; 5 Centre for Policy Impact in Global Health, Duke University, Durham, North Carolina; 6 Duke Global Health Institute, Duke University, Durham, North Carolina; 7 Bergen Centre for Ethics and Priority Setting (BCEPS), Department of Global Public Health and Primary Care, University of Bergen, Bergen, Norway; 8 Department of Global Health and Population, Harvard T.H. Chan School of Public Health, Boston, Massachusetts; 9 Danish Institute for Advanced Study, University of Southern Denmark, Copenhagen, Denmark; 10 Danish Centre for Health Economics, University of Southern Denmark, Odense, Denmark; 11 Department of Population Health Sciences, Duke School of Medicine, Duke University, Durham, North Carolina

## Abstract

**Importance:**

Persistent disparities in mortality across countries suggest uneven improvements in living standards and access to life-extending health technologies, as well as context-specific obstacles. Studies have analyzed cross-country inequality in mortality but have not widely contextualized those disparities in terms of developmental progress relative to a frontier representing a level of mortality achievable with broad access to the best health-enhancing technology and living standards available.

**Objective:**

To examine probability of premature death (PPD)—defined as probability of dying before 70 years of age—across countries and regions, benchmarking progress as years behind the lowest country-level PPD (the frontier).

**Design and Setting:**

This cross-sectional study used aggregate-level data from the 2024 United Nations World Population Prospects and Human Mortality Database to calculate PPD across 7 global regions and the 30 most populous countries. Data were analyzed from May to September 2025.

**Main Outcome and Measures:**

The primary outcomes were PPD and the number of years behind the lowest country-level PPD.

**Results:**

The frontier PPD fell from 57% to 12% from 1900 to 2019. Sub-Saharan Africa’s PPD in 2019 was 52%, corresponding to the 1916 frontier PPD. However, sub-Saharan Africa had converged toward the frontier by over 40 years since 2000, when it had a 65% PPD. China has been converging toward the frontier since 1970, having been 93 years behind the frontier PPD in 1970 (with a 60% PPD) and 35 years behind in 2019 (21% PPD). The US has diverged away from the frontier, having been 29 years behind in 1970 (38% PPD) and 38 years in 2019 (22% PPD). Of the regions included, the North Atlantic (Western Europe and Canada) was the closest to the frontier, being 13 years behind in 2019 (15% PPD). The US, Central and Eastern Europe, and sub-Saharan Africa were the furthest above the 2019 PPD Preston curve (ie, they had a greater PPD than predicted by their per capita gross domestic product).

**Conclusions and Relevance:**

In this cross-sectional study, disparities in PPD were likely to reflect major inequality in access to health-enhancing technologies and living standards, as well as context-specific obstacles. Technological and medical advancements leading to universal health benefits need to be rapidly and fairly disseminated.

## Key Points

**Question:**

How does the probability of premature death (PPD)—defined as probability of dying before 70 years of age—in 7 global regions and the 30 most populous countries compare to a frontier representing the lowest country-level PPD?

**Findings:**

In this cross-sectional study, the frontier PPD fell from 57% to 12% from 1900 to 2019. Of the 7 global regions highlighted here, the greatest convergence toward the frontier since 2000 was observed in sub-Saharan Africa, which had the same PPD as the frontier almost 150 years earlier in the year 2000 and just over 100 years earlier in 2019.

**Meaning:**

This study underscores persistent inequality in living standards and access to health-enhancing technologies, as well as context-specific obstacles that can impact long-run declines in PPD.

## Teaser

This cross-sectional study examines probability of premature death across countries and regions and shows their years behind or ahead of the 2019 Preston curve.

## Introduction

Life expectancy has been on an upward trajectory for 2 centuries, thanks to advances in medical science, public health, and living standards.^1,2,3^ However, progress has been uneven, leading to stark disparities in mortality across regions and countries. For example, in 2019, the probability of premature death (PPD)—the chance of dying before 70 years of age—was 15% in Western Europe and Canada, 22% in the US, 37% in India, and 52% in sub-Saharan Africa.^4^

Development is a long-run process, and gaps in important outcomes such as PPD across countries highlight unequal improvements of living standards and access to life-extending health technologies, as well as context-specific obstacles.^5,6,7^ The frontier represents the lowest county-level PPD demonstrated to be achievable and reflects broad access to the best health-enhancing technology and living standards available at each point in time. This comparative analysis shows how many years regions and countries were behind the frontier PPD (ie, the lowest country-level PPD each year). For instance, if a region had a PPD in 2019 that was the same as the frontier PPD in 1989, we considered that region to be 30 years behind regarding the best PPD achievable. We show development from 1970 to 2019 to explore whether regions are converging toward or diverging away from the frontier PPD.

## Methods

### Data Sources

Age-specific mortality probabilities from a period life table were used to calculate the probability of dying before age 70 years (*_70_q_0_*), or PPD, the main outcome used by the *Lancet* Commission on Investing in Health. Mortality probabilities for single-year age intervals were obtained for countries and territories between 1950 and 2023 from the United Nations World Population Prospects (UN WPP) 2024 life tables.^8^ These were used to construct PPD for each region and the 30 most populous countries from 1970 to 2019, as well as the frontier PPD from 1950 to 2019. Since regions and countries were often further behind the frontier PPD than 1950, we supplemented the UN WPP data with data from the Human Mortality Database (HMD)^9^ for constructing the frontier PPD, which makes it possible to extend it back to 1751.

The early HMD estimates were only available for a few countries; however, these now high-income countries (eg, Sweden, Norway, the Netherlands) were likely to be (or at least close to being) the global frontier before 1950. For example, Norway was the frontier in the HMD data from 1945 to 1950 and remained the frontier from 1950 to 1962 according to the UN data (eTable 1 in Supplement 1). Norway, Sweden, or the Netherlands were the frontier from 1900 to 1976, when they were overtaken by Japan, which remained the frontier until 2010, when it was overtaken by South Korea or Italy some years.

We restricted the main analyses to years before 2020 to avoid (presumably) temporary distortions due to the COVID-19 pandemic. We followed the regional classification of the *Lancet* Commission on Investing in Health,^10^ highlighting results for 7 global regions and the 3 most populous countries: China, India, and the US (eTable 2 in Supplement 1). We also show results for the 30 most populous countries (in 2019),^8^ for 2023, and by sex.

This project used publicly accessible aggregate data from the UN WPP, HMD, and Maddison Project. These activities do not meet the regulatory definition of human participant research. As such, institutional review board approval and patient informed consent were not required in accordance with the Common Rule (45 CFR §46). We followed the Strengthening the Reporting of Observational Studies in Epidemiology (STROBE) reporting guidelines.

### Years Behind the Frontier PPD

The frontier PPD was defined as the country with the lowest PPD each year. We excluded countries with a population below 5 million in 2019 and countries where a very large share of the population were migrants (specifically United Arab Emirates, Hong Kong, and Switzerland), who may have better health outcomes due to healthy-migrant effects (which suggests migrants are on average healthier than nonmigrants) and salmon bias (which suggests migrants return home prior to death).^11^ The exclusion of United Arab Emirates, Hong Kong, and Switzerland only changed the frontier slightly and only for a few recent years and, therefore, has no large impact on the overall result.

Since life-extending technologies generally only advance, we considered increases in PPD across years to be temporary setbacks (eg, due to high prevalence of smoking across high-income countries^12^ or pandemics). Therefore, we removed increases in PPD across years, so the frontier is the lowest country-level PPD ever observed up until and including each year. In other words, the interpretation of the frontier is the lowest PPD demonstrated to be possible with the most advanced technology available each year. The frontiers from 1900 to 2019 are shown in eTable 1 in Supplement 1. While large temporary dips in the lowest PPD each year may distort the frontier somewhat (since the bottom of the dip becomes the frontier PPD until a lower PPD is reached again), this is mainly an issue before 1900 (eFigure 1 in Supplement 1).

We determined how many years behind a region or country was compared with the frontier. As an example, consider India in 2019, which had a PPD of 37%. We identified the most recent year that the frontier PPD was greater than or equal to India’s 2019 PPD. That year was 1944. Therefore, in 2019, India was 75 years behind the frontier. We did the same for each region and country from 1970 to 2019.

### Estimating Years Behind or Ahead of Preston Curves

The Preston curve is a seminal concept relating cross-sectionally life expectancy to per capita aggregate income.^13^ With technological advancements, the Preston curve is expected to shift upward across time, such that the same levels of income would result in better life expectancy in more recent years (eAppendix 1 in Supplement 1).^14,15^ This shift allowed us to estimate on which year’s Preston curve countries and regions lay. For this analysis, we obtained real per capita gross domestic product (GDP; in 2011 international dollars), available for 169 countries from the 2023 version of the Maddison Project database.^16^ We first estimated Preston curves for PPD (instead of life expectancy, as is conventionally done) for each year between 1950 and 2019 to use as benchmarks. Note that the curve is expected to shift downward across time when the outcome is PPD rather than life expectancy. In short, we regressed PPD on log per capita GDP and intercepts for each year between 1950 and 2019, including 169 countries (few countries had missing data for some years), with countries equally weighted (eAppendix 2 in Supplement 1).

First, we determined whether the PPD observed in a region in 2019 was higher or lower than the PPD predicted by that region’s 2019 per capita GDP. In other words, we determined whether a region was above (behind) or below (ahead) the 2019 Preston curve. The region was behind the Preston curve in 2019 if its observed PPD was greater than the PPD predicted from the Preston curve using that region’s 2019 per capita GDP—meaning that the region had a higher PPD than expected given its level of income. Then, we identified the most recent Preston curve where the target region’s 2019 per capita GDP predicted a PPD greater than or equal to the PPD observed in that region in 2019. Conversely, the region was ahead of the Preston curve in 2019 if its observed PPD was lower than the PPD predicted from the 2019 Preston curve using that region’s 2019 per capita GDP. Then, we identified the most recent year the target region’s PPD was equal to or greater than the PPD predicted by the target region’s 2019 GDP and the 2019 Preston curve.

### Sensitivity Analyses

Although PPD decline appeared to be fairly linear from 1880 to 2019, there were periods where PPD decline slowed, stagnated, or accelerated, meaning the relationship between PPD and years behind is not constant across levels of PPD (eFigure 1 in Supplement 1). Notably, the frontier stagnated at 28% PPD from 1956 to 1965, which induced a sharp reduction in years behind over periods when PPD declined below 28% in regions and countries. As a sensitivity analysis, we estimated a linear trend for the frontier from 1820 to 2019 and used it as a benchmark. We did not use this linear frontier in the main analyses since changes in the rate of decline in the frontier may imply true changes in the advancement of health-enhancing technologies. All analyses were conducted using Stata, version 16 (StataCorp), and data were analyzed from May to September 2025.

## Results

### Frontier PPD and PPD for Regions in 2019

**Figure 1.**
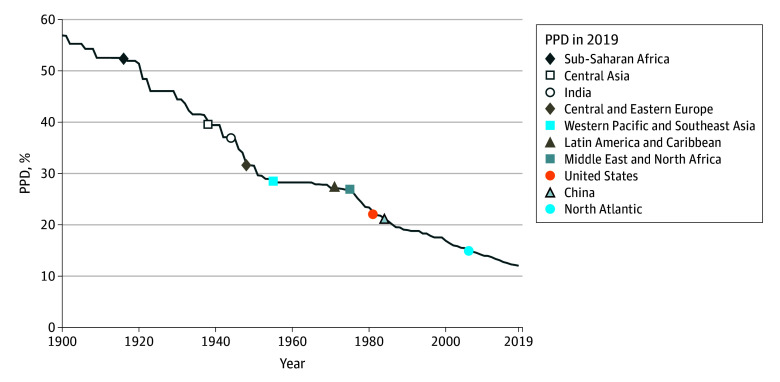
Frontier Probability of Premature Death (PPD) Across Time and for Regions in 2019 PPD was defined as dying before 70 years of age. The line shows frontier PPD each year, while the markers indicate PPD in 2019 for each location. The frontier is the lowest PPD ever observed up until and including each year (ie, increases across years were removed). Countries with a population below 5 million in 2019 were not considered for being a frontier.

The frontier PPD fell from 57% to 12% from 1900 to 2019 (Figure 1). Sub-Saharan Africa was the furthest behind the frontier, with 52% PPD in 2019, which was last observed in the frontier in 1916, putting sub-Saharan Africa 103 years behind the frontier. Central Asia was 81 years behind (with 40% PPD), India was 75 years behind (37% PPD), Central and Eastern Europe were 71 years behind (32% PPD), Latin America and the Caribbean were 48 years behind (27% PPD), the Middle East and North Africa were 44 years behind (27% PPD), the US was 38 years behind (22% PPD), China was 35 years behind (21% PPD), and the North Atlantic (Western Europe and Canada) was 13 years behind (15% PPD).

### Years Behind Frontier PPD Across Time

**Figure 2.**
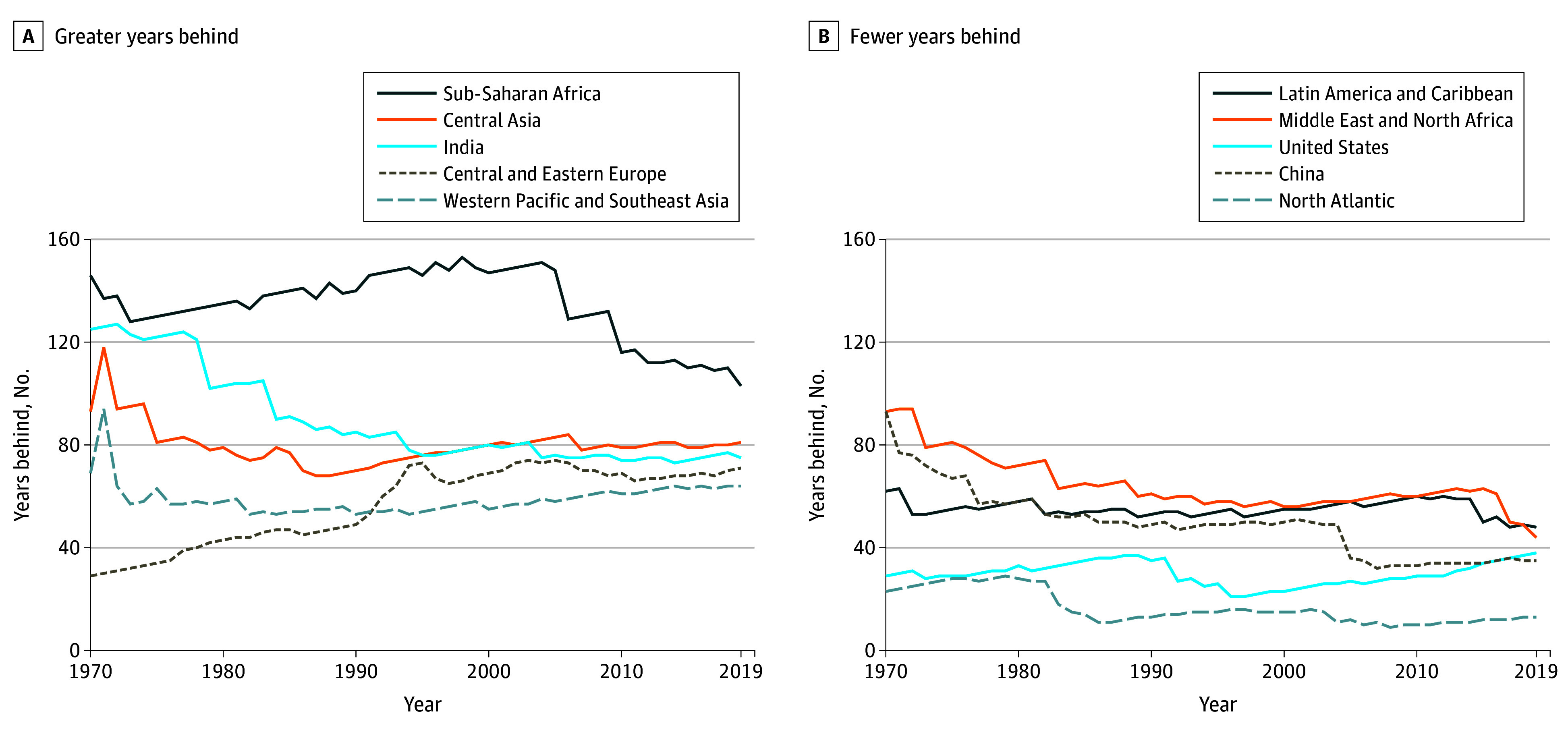
Years Behind the Frontier Probability of Premature Death (PPD), 1970-2019 PPD was defined as dying before 70 years of age. The frontier is the lowest PPD ever observed up until and including each year (ie, increases across years were removed). Countries with a population below 5 million in 2019 were not considered for being a frontier.

In 1970, sub-Saharan Africa had a 72% PPD, equivalent to the frontier PPD in 1824 (Figure 2). Sub-Saharan Africa first converged toward the frontier, being 128 years behind in 1973 (70% PPD), then diverged until around 2000, when it was 147 years behind (65% PPD), after which it converged substantially toward the frontier. Central Asia converged somewhat toward the frontier PPD between 1971 and around 1988, from being 118 years behind (65% PPD) to being 68 years behind (51% PPD). Central Asia then diverged from the frontier most years from 1988 to 2006 and remained largely unchanged relative to the frontier from 2006 to 2019.

India converged substantially toward the frontier between 1970 and around 2014, from being 125 years behind (68% PPD) to being 73 years behind (39% PPD). In 1970, Central and Eastern Europe were 29 years behind (39% PPD) and diverged away from the frontier PPD until around 1995, when they were 73 years behind (47% PPD), then remaining largely unchanged relative to the frontier. Western Pacific and Southeast Asia remained largely unchanged relative to the frontier PPD from 1970 to 2019, going from being 69 years behind (56% PPD) to being 64 years behind (29% PPD), converging slightly between 1970 and 1976, and diverging again after around 2000. Latin America and the Caribbean remained largely unchanged relative to the frontier, being 53 years behind in 1972 (52% PPD), diverging slightly until 2012—when they were 60 years behind (29% PPD)—then converging from 2012 to 2019. The Middle East and North Africa converged between 1970 and around 1994, from being 93 years behind (61% PPD) to being 57 years behind (41% PPD), then diverging slightly until 2015—when they were 63 years behind (29% PPD)—and converging sharply until 2019 (coinciding with a stagnation in the frontier PPD at 28% in from 1956 to 1965).

The US diverged between 1970 and around 1989, from being 29 years behind (38% PPD) to 37 years behind (29% PPD), then converged sharply (coinciding with the stagnation in the frontier PPD at 28% in 1956), being 21 years behind (27% PPD) in 1996. The US then diverged steadily from around 1997 to 2019. China converged steadily from 1970 to around 1986, going from being 93 years behind (60% PPD) to being 50 years behind (41% PPD). China converged sharply toward the frontier in 2005 (coinciding with the stagnation in the frontier PPD at 28% in 1956), when it went from being 49 years to 36 years behind. The North Atlantic was the closest to the frontier, being 23 years behind (34%) in 1970, 29 years behind (30% PPD) in 1979, 11 years behind (26% PPD) in 1987, 16 years behind (22% PPD) in 1997, 9 years behind (17% PPD) in 2008, and 13 years behind (15% PPD) in 2019.

**Figure 3.**
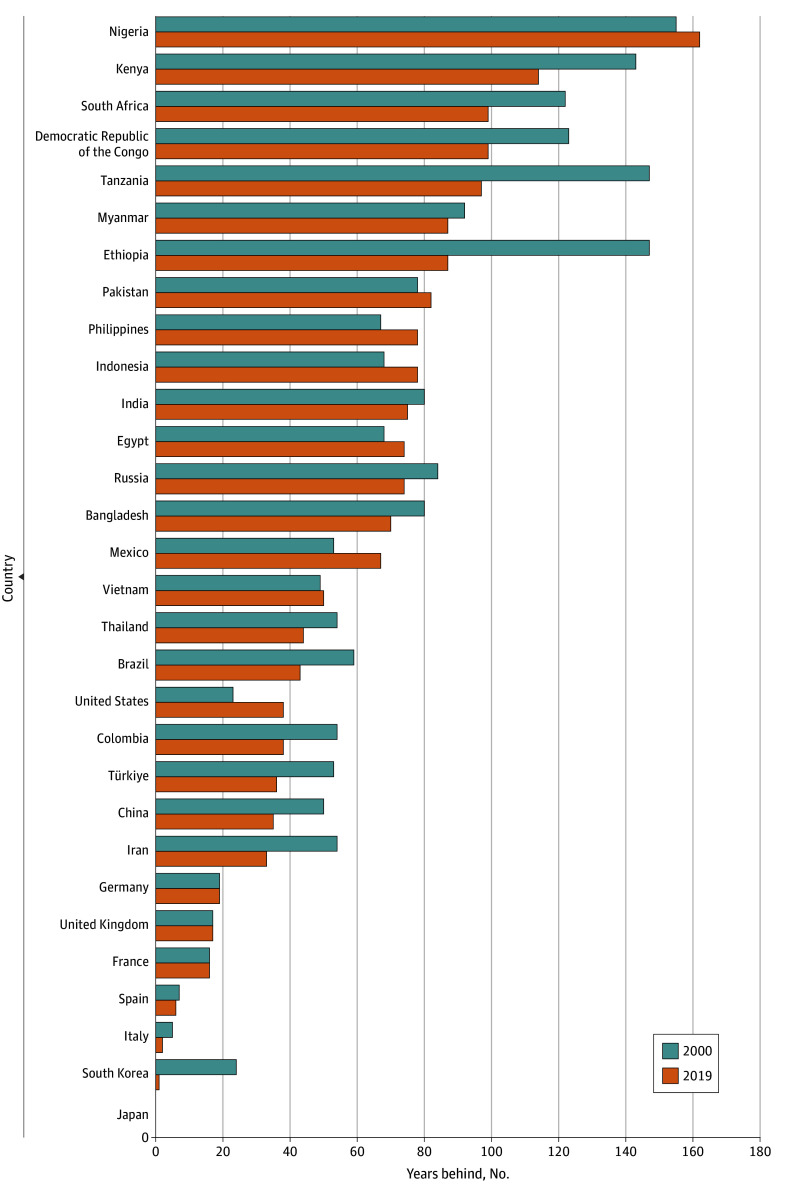
Years Behind the Frontier Probability of Premature Death (PPD) in 2000 and 2019 Among the 30 Most Populous Countries PPD was defined as dying before 70 years of age. The frontier is the lowest PPD ever observed up until and including each year (ie, increases across years were removed). Countries with a population below 5 million in 2019 were not considered for being a frontier.

Among the 30 largest countries, Japan, South Korea, Italy, and Spain were the closest to the frontier PPD in 2019 (in fact, Japan was the frontier [Figure 3]). Nigeria was the furthest behind, both in 2000 and 2019. A majority of regions and countries moved further away from the frontier in 2023 (eFigures 2 and 3 in Supplement 1).

**Figure 4.**
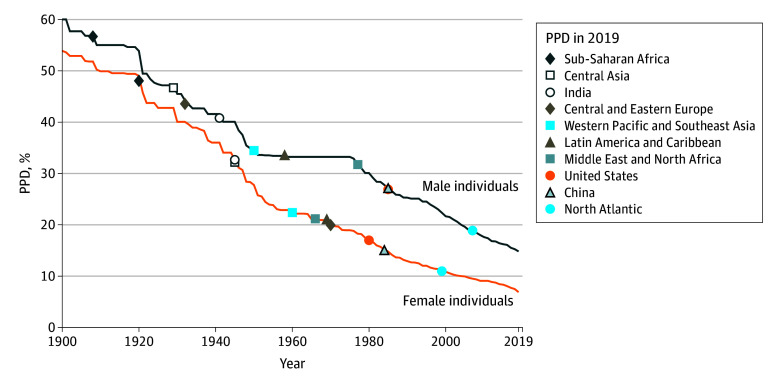
Frontier Probability of Premature Death (PPD) Across Time and for Regions in 2019 by Sex PPD was defined as dying before 70 years of age. The line shows frontier PPD each year, while the markers indicate PPD in 2019 for each location. The frontier is the lowest PPD ever observed up until and including each year (ie, increases across years were removed). Countries with a population below 5 million in 2019 were not considered for being a frontier.

The male frontier had greater PPD than the female frontier throughout the period (Figure 4). Furthermore, the male frontier stagnated from around 1955 to 1975 (when the lowest country-level PPD each year increased across years [eTable 1 in Supplement 1]), while the female frontier declined. Still, male individuals in Central and Eastern Europe were much further away from the male frontier (87 years) than female individuals were from the female frontier (49 years). The patterns were overall similar when using a linear frontier (eFigures 4-7 in Supplement 1).

### Behind or Ahead of the Preston Curve

**Figure 5.**
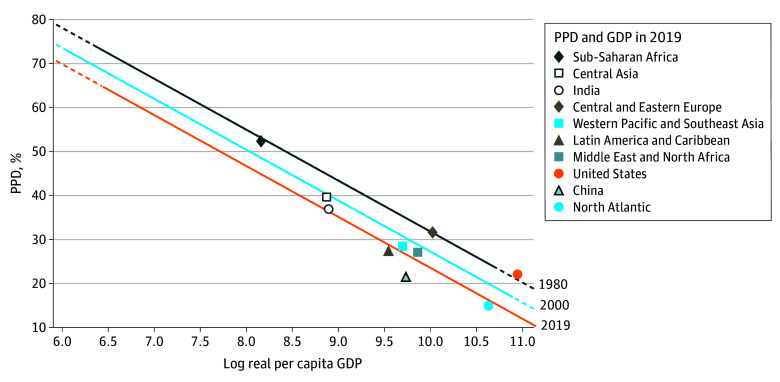
Preston Curves for 1980, 2000, and 2019 and Observed Probability of Premature Death (PPD) and Gross Domestic Product (GDP) for Regions in 2019 PPD was defined as dying before 70 years of age. Markers indicate PPD and GDP (in 2011 international dollars) for each region in 2019. The dashed lines indicate GDP beyond what was observed in that year. Preston curves were estimated for each year by regressing PPD on log of GDP with a separate intercept for each year (unweighted). The slope for GDP is constant across years, while the intercept varies across years. The intercepts were adjusted such that they never increased across years.

The US, Central and Eastern Europe, sub-Saharan Africa, Central Asia, the Middle East and North Africa, Western Pacific and Southeast Asia, and India were all above the 2019 Preston curve (Figure 5). That is, they had a greater PPD than predicted by their per capita GDP. For example, in 2019, India had a per capita GDP of approximately $7300 and a PPD of 37%. Meanwhile, the 2019 Preston curve predicted a PPD of 36% for a $7300 per capita GDP; therefore, India was behind (or above) the Preston curve in 2019. The most recent Preston curve to predict a PPD at or above 37% for a per capita GDP of $7300 was in 2016, putting India 3 years behind the 2019 Preston curve. The US was the furthest behind, considering its per capita GDP, being on the 1976 Preston curve in 2019. Meanwhile, Central and Eastern Europe were on the 1979 Preston curve, sub-Saharan Africa was on the 1981 curve, and Central Asia was on the 2008 curve. Western Pacific and Southeast Asia were 7 years behind, and the Middle East and North Africa were 6 years behind the Preston curve.

Other regions were ahead of the Preston curve. For example, in 2019, China had a per capita GDP of almost $17 000 and a PPD of 21%. The 2019 Preston curve predicted 27% PPD for $17 000 per capita GDP. The most recent year China had a PPD above 27% was 2006, putting China 13 years ahead of the Preston curve in 2019. The North Atlantic was 8 years ahead, and Latin America and the Caribbean were 7 years ahead. Using a more flexible specification when predicting PPD for a given GDP showed largely similar results as using Preston curves with fixed slopes across years (eFigure 8 in Supplement 1).

## Discussion

The frontier PPD fell from 57% to 12% from 1900 to 2019, reflecting nearly uninterrupted gains in living standards and health-enhancing technologies. Progress has been uneven across regions and countries. In 2019, sub-Saharan Africa was 103 years behind the frontier, Central Asia was 81 years behind, and India was 75 years behind, which may reflect a wide range of factors, such as difficulties in implementing and accessing health-enhancing innovation, low living standards, and unique challenges related to these contexts.

However, Central Asia, India, and especially sub-Saharan Africa converged toward the frontier PPD between 1970 and 2019. For example, sub-Saharan Africa was 147 years behind in 2000 compared to 103 years in 2019. China converged toward the frontier PPD from 1970 to 2019 (from being 93 years to 35 years behind), while the US diverged (from being 29 years to 38 years behind), especially after 2010. In fact, China was closer to the frontier PPD than the US in 2019. Of the global regions highlighted herein, the North Atlantic was the closest to the frontier PPD in 2019, being 13 years behind. From the 30 most populous countries, Japan was the frontier in 2019 and was followed by South Korea.

Despite enormous improvements, sub-Saharan Africa continues to have high mortality from infectious diseases and neonatal and maternal conditions.^17,18^ Although infectious diseases and neonatal conditions continue to play a sizable role in premature mortality in Central Asia and India, noncommunicable diseases are increasingly driving premature mortality differentials, suggesting a need to control risk factors and apply policy tools to tackle early deaths from cardiovascular disease (eg, by controlling smoking, hypertension, and high cholesterol).^17^

Early advances in agricultural technology improved nutrition,^19^ and discovery of the germ theory of diseases eventually led to improvements in sanitation, hygiene, and food standards, causing early declines in mortality and increased life expectancy in today’s high-income countries in the late 19th and early 20th centuries.^20,21^ Implementing these early technological innovations required coordinated public effort and major investments into large-scale projects. Health improvements were, therefore, somewhat more contingent on economic growth. Over time, these costs have gone down. Also, in the later 20th century, health-enhancing innovations were to a larger extent individually targeted medical interventions. For example, vaccines, oral rehydration therapy, and ACE inhibitors can have large health benefits at a low cost,^22^ enabling countries to achieve better health at a lower-level economic development than before. Therefore, the Preston curve has shifted, such that the same level of per capita GDP affords better health today than in the past. When considering the PPD expected for a given per capita GDP using Preston curves, sub-Saharan Africa, Central and Eastern Europe, and the US were furthest behind (above) the 2019 PPD Preston curve. On the other hand, China was the further ahead (below) the 2019 PPD Preston curve.

The structural, historical, and sociopolitical context of each region plays a crucial role in shaping health outcomes at any level of economic development. Incomes may be unequally distributed, not spent on health, or even achieved through means that are detrimental to health. Conversely, countries can also make use of resources efficiently, prioritize health, implement cost-effective interventions, or practice healthy behaviors (eg, healthier diet and alcohol restrictions) and thereby achieve low mortality at low level of GDP.^23,24^ However, ultimately, aggregate income constrains living standards and the ability to implement effective health interventions—such as health care, public safety, and establishment and maintenance of water, sanitation, and other important infrastructure.

The reasons economic development does not translate into low mortality are likely to vary across regions. Despite a high per capita GDP (and the highest global health care spending^25^), the US has high levels of economic inequality.^26^ The individual income-health relationship tends to show diminishing marginal returns in health to increased income; therefore, greater inequality at the same level of aggregate income will lead to higher observed mortality.^27,28,29^ Furthermore, the health care systems in the US suffer from spending waste^30^ and gun violence (homicide and suicide), and road traffic deaths cause an unusually high loss of life for a high-income country.^17,18^ The US is also going through a substance use epidemic.^31^ In fact, despite continued economic growth, life expectancy in the US has been declining.^32^

Central and Eastern Europe have witnessed rapid economic growth, especially since 1990, but continue to have a large number of deaths due to alcohol misuse and suicide, especially among male individuals.^18,33^ The prominence of the mining sector in sub-Saharan Africa and the resource curse—suggested to reduce human development by decreasing equality,^34^ quality of institutions,^35^ and public spending^36^—may to an extent explain why it remains far behind, both in general and relative to aggregate income.^37^ The HIV/AIDS epidemic, and a delayed response to that epidemic, has limited convergence and even set sub-Saharan Africa further behind the frontier initially, before making impressive progress from 2000 to 2019.

### Limitations

This study has several limitations. First, the conclusions rely on the quality and availability of mortality data across regions. However, we relied on estimates from the 2024 UN WPP and HMD for the main analysis, which are widely used. Second, comparing regions based on years behind the frontier PPD progress simplifies complex temporal dynamics and regional disparities. The frontier PPD also did not decline at a constant rate; therefore, a single percentage-point decline in PPD may lead to vastly different changes in years behind depending on the level of PPD. Third, the associations between per capita GDP and PPD estimated in this study were not causal. However, our goal was to describe expected PPD for a given level of economic development, which does not imply causality. Fourth, the analyses were purely descriptive and do not provide explanations for the differences observed. Fifth, we used a period measure of mortality while improvements in health-enhancing technologies and living standards have a somewhat delayed impact.

## Conclusions

This cross-sectional study shows that in the context of long run and largely uninterrupted declines in premature mortality, the present comparative analytic approach illuminates uneven progress across regions. By comparing mortality disparities as years behind a frontier, this study highlights the temporal nature of mortality declines across the world.

## Notes

Supplement 1. **eTable 1.** PPD Frontier **eTable 2.** Regions **eAppendix 1. **Economic Growth and Mortality **eAppendix 2.** Estimating Preston Curves **eFigure 1. **Lowest PPD, Frontier PPD, and Linear Prediction of Frontier PPD 1820-2019 **eFigure 2. **Years Behind Frontier PPD in 2019 and 2023: Regions **eFigure 3. **Years Behind Frontier PPD in 2019 and 2023: 30 Most Populous Countries **eFigure 4. **Linear Frontier PPD Across Time (Line) and for Regions in 2019 (Markers) **eFigure 5. **Years Behind Linear Frontier PPD 1970-2019 **eFigure 6. **Years Behind Linear Frontier PPD in 2000 and 2019: 30 Most Populous Countries **eFigure 7.** Linear Frontier PPD Across Time (Line) and for Regions in 2019 (Markers): By Sex **eFigure 8.** Years Behind or Ahead of PPD Expected for Level of Economic Development
eReferences

Supplement 2.
Data Sharing Statement
