# Supplementary material for: Global Disparities in Premature Mortality
Source: JAMA Health Forum. 2025 Oct 3;6(10):e253479. doi: 10.1001/jamahealthforum.2025.3479 (PMC12495499; doi:10.1001/jamahealthforum.2025.3479)
Supplement: Supplement 1. — eTable 1. PPD Frontier eTable 2. Regions eAppendix 1. Economic Growth and Mortality eAppendix 2. Estimating Preston Curves eFigure 1. Lowest PPD, Frontier PPD, and Linear Prediction of Frontier PPD 1820-2019 eFigure 2. Years Behind Frontier PPD in 2019 and 2023: Regions eFigure 3. Years Behind Frontier PPD in 2019 and 2023: 30 Most Populous Countries eFigure 4. Linear Frontier PPD Across Time (Line) and for Regions in 2019 (Markers) eFigure 5. Years Behind Linear Frontier PPD 1970-2019 eFigure 6. Years Behind Linear Frontier PPD in 2000 and 2019: 30 Most Populous Countries eFigure 7. Linear Frontier PPD Across Time (Line) and for Regions in 2019 (Markers): By Sex eFigure 8. Years Behind or Ahead of PPD Expected for Level of Economic Development eReferences [file jamahealthforum-e253479-s001.pdf]

Supplemental Online Content

Karlsson O, Jamison DT, Yamey G, et al. Global disparities in premature mortality. *JAMA Health Forum*. 2025;6(10):e253479. doi:10.1001/jamahealthforum.2025.3479

eTable 1. PPD Frontier

eTable 2. Regions

eAppendix 1. Economic Growth and Mortality

eAppendix 2. Estimating Preston Curves

eFigure 1. Lowest PPD, Frontier PPD, and Linear Prediction of Frontier PPD 1820-2019

eFigure 2. Years Behind Frontier PPD in 2019 and 2023: Regions

eFigure 3. Years Behind Frontier PPD in 2019 and 2023: 30 Most Populous Countries

eFigure 4. Linear Frontier PPD Across Time (Line) and for Regions in 2019 (Markers)

eFigure 5. Years Behind Linear Frontier PPD 1970-2019

eFigure 6. Years Behind Linear Frontier PPD in 2000 and 2019: 30 Most Populous Countries

eFigure 7. Linear Frontier PPD Across Time (Line) and for Regions in 2019 (Markers): By Sex

eFigure 8. Years Behind or Ahead of PPD Expected for Level of Economic Development

eReferences

This supplemental material has been provided by the authors to give readers additional information about their work.

© 2025 Karlsson O et al. *JAMA Health Forum*.

eTable 1. PPD Frontier

	Both sexes				Males				Females			
	Frontier (lowest ever)		Lowest PPD for year		Frontier (lowest ever)		Lowest PPD for year		Frontier (lowest ever)		Lowest PPD for year	
Year	Country	PPD, %	Country	PPD, %	Country	PPD, %	Country	PPD, %	Country	PPD, %	Country	PPD, %
1900	Norway	56.90	Norway	59.02	Norway	60.02	Norway	61.91	Norway	53.90	Norway	56.21
1901	Norway	56.83			Norway	60.02	Norway	60.14	Norway	53.61		
1902	Norway	55.27			Norway	57.72			Norway	52.91		
1903	Norway	55.27	Sweden	55.94	Norway	57.72	Sweden	58.94	Norway	52.91	Sweden	53.11
1904	Norway	55.27	Norway	55.85	Norway	57.72	Norway	58.09	Norway	52.91	Denmark	53.45
1905	Norway	55.27	Norway	57.02	Norway	57.72	Norway	59.27	Norway	52.91	Norway	54.94
1906	Norway	54.29			Norway	56.84			Norway	51.91		
1907	Norway	54.29	Sweden	54.48	Norway	56.84	Sweden	57.33	Sweden	51.82		
1908	Norway	54.29	Sweden	55.10	Norway	56.84	Norway	57.61	Sweden	51.82	Sweden	52.64
1909	Sweden	52.54			Sweden	55.03			Sweden	50.23		
1910	Sweden	52.54	Sweden	53.34	Sweden	55.03	Sweden	56.12	Denmark	49.91		
1911	Norway	52.54	Norway	53.01	Sweden	55.03	Norway	56.29	Denmark	49.91	Norway	49.93
1912	Norway	52.54	Sweden	53.65	Sweden	55.03	Sweden	56.58	Denmark	49.91	Sweden	50.87
1913	Denmark	52.53			Denmark	55.03	Denmark	55.31	Sweden	49.56		
1914	Denmark	52.53	Sweden	52.89	Denmark	55.03	Sweden	55.53	Sweden	49.56	Denmark	49.70
1915	Denmark	52.53	Norway	53.43	Denmark	55.03	Denmark	56.38	Sweden	49.56	Norway	50.04
1916	Sweden	52.53	Sweden	52.69	Sweden	55.03	Sweden	55.28	Sweden	49.56	Sweden	50.27
1917	Sweden	51.95			Sweden	54.65			Sweden	49.42		
1918	Sweden	51.95	Denmark	55.99	Sweden	54.65	Denmark	57.58	Sweden	49.42	Denmark	54.46
1919	Denmark	51.95	Denmark	55.03	Denmark	54.65	Denmark	56.17	Sweden	49.42	Sweden	52.52
1920	Sweden	51.40			Sweden	53.87			Sweden	49.02		
1921	Norway	48.43			Denmark	49.46			Norway	45.77		
1922	Norway	48.43	Australia	49.29	Denmark	49.46	Sweden	50.83	Australia	43.75		
1923	Sweden	46.07			Sweden	48.38			Australia	43.75	Sweden	43.90
1924	Sweden	46.07	Netherlands	46.91	Netherlands	47.69			Australia	43.75	Australia	44.29
1925	Netherlands	46.07	Netherlands	46.39	Netherlands	47.39			Australia	42.78		
1926	Netherlands	46.07	Netherlands	46.43	Netherlands	47.19			Australia	42.78	Australia	43.86
1927	Netherlands	46.07	Norway	47.14	Netherlands	47.19	Netherlands	48.13	Australia	42.78	Australia	44.11
1928	Netherlands	46.07	Netherlands	46.29	Netherlands	47.19	Netherlands	47.36	Norway	42.78	Norway	43.86
1929	Netherlands	46.07	Norway	47.13	Netherlands	47.19	Denmark	49.29	Australia	42.78	Australia	43.03

© 2025 Karlsson O et al. *JAMA Health Forum.*

	Both sexes				Males				Females			
	Frontier (lowest ever)		Lowest PPD for year		Frontier (lowest ever)		Lowest PPD for year		Frontier (lowest ever)		Lowest PPD for year	
Year	Country	PPD, %	Country	PPD, %	Country	PPD, %	Country	PPD, %	Country	PPD, %	Country	PPD, %
1930	Netherlands	44.44			Netherlands	45.50			Australia	40.07		
1931	Netherlands	44.44	Norway	44.94	Netherlands	45.50	Netherlands	46.50	Australia	40.07	Australia	40.20
1932	Netherlands	43.65			Netherlands	44.25			Australia	40.07	Australia	40.07
1933	Norway	42.25			Netherlands	43.44			Norway	39.55		
1934	Netherlands	41.53			Netherlands	42.69			Norway	38.93		
1935	Netherlands	41.53	Netherlands	41.92	Netherlands	42.69	Netherlands	43.04	Norway	38.93	Norway	39.34
1936	Netherlands	41.53	Netherlands	41.54	Netherlands	42.69	Netherlands	42.81	Norway	38.59		
1937	Netherlands	41.39			Netherlands	42.68			Norway	38.31		
1938	Norway	40.23			Netherlands	41.59			Norway	36.45		
1939	Norway	39.44			Netherlands	41.59	Netherlands	41.62	Norway	36.03		
1940	Norway	39.44	Sweden	41.35	Netherlands	41.59	Denmark	44.25	Norway	36.03	Norway	36.89
1941	Sweden	39.44	Sweden	40.32	Sweden	41.59	Sweden	42.99	Norway	36.03	Norway	36.55
1942	Sweden	37.07			Sweden	40.07			Sweden	34.06		
1943	Sweden	37.07	Sweden	37.58	Sweden	40.07	Sweden	40.43	Sweden	34.06	Sweden	34.73
1944	Sweden	37.07	Sweden	39.16	Sweden	40.07	Sweden	42.45	Sweden	34.06	Norway	35.32
1945	Norway	36.85			Sweden	40.07	Sweden	40.48	Norway	32.75		
1946	Norway	34.75			Norway	38.40			Norway	31.14		
1947	Norway	34.07			Norway	37.53			Norway	30.69		
1948	Norway	31.90			Norway	35.46			Norway	28.39		
1949	Norway	31.60			Norway	34.92			Norway	28.34		
1950	Norway	31.52			Netherlands	34.89			Norway	27.76		
1951	Norway	29.65			Norway	33.61			Norway	25.75		
1952	Norway	29.55			Norway	33.61	Norway	33.66	Norway	25.53		
1953	Norway	28.96			Norway	33.54			Norway	24.43		
1954	Norway	28.94			Norway	33.54	Norway	34.00	Norway	23.94		
1955	Norway	28.63			Norway	33.44			Norway	23.84		
1956	Norway	28.26			Norway	33.44	Norway	33.51	Norway	23.05		
1957	Norway	28.26	Norway	28.54	Norway	33.44	Netherlands	34.16	Norway	22.88		
1958	Norway	28.26	Norway	28.63	Sweden	33.44	Sweden	33.99	Norway	22.88	Norway	23.02
1959	Norway	28.26	Sweden	28.77	Sweden	33.24			Norway	22.88	Norway	23.46
1960	Norway	28.26	Norway	28.66	Sweden	33.24	Norway	34.47	Norway	22.75		
1961	Norway	28.26	Norway	28.67	Sweden	33.24	Sweden	33.98	Norway	22.18		
1962	Norway	28.26	Sweden	29.00	Sweden	33.24	Sweden	34.62	Norway	22.18	Norway	22.36

© 2025 Karlsson O et al. *JAMA Health Forum*.

	Both sexes				Males				Females			
	Frontier (lowest ever)		Lowest PPD for year		Frontier (lowest ever)		Lowest PPD for year		Frontier (lowest ever)		Lowest PPD for year	
Year	Country	PPD, %	Country	PPD, %	Country	PPD, %	Country	PPD, %	Country	PPD, %	Country	PPD, %
1963	Sweden	28.26	Sweden	28.76	Sweden	33.24	Sweden	34.05	Norway	22.18	Norway	22.63
1964	Sweden	28.26	Sweden	28.68	Sweden	33.24	Sweden	34.25	Norway	22.11		
1965	Sweden	28.25			Sweden	33.24	Sweden	33.87	Norway	21.11		
1966	Sweden	27.89			Sweden	33.24	Sweden	34.03	Norway	21.11	Norway	21.22
1967	Sweden	27.89	Sweden	27.90	Sweden	33.24	Sweden	34.18	Norway	20.94		
1968	Sweden	27.81			Sweden	33.24	Sweden	33.88	Norway	20.94	Norway	21.01
1969	Sweden	27.81	Sweden	28.04	Sweden	33.24	Sweden	34.41	Norway	20.94	Sweden	21.46
1970	Sweden	27.19			Sweden	33.24	Sweden	33.39	Norway	20.10		
1971	Sweden	27.19	Sweden	27.31	Sweden	33.24	Sweden	33.94	Norway	19.72		
1972	Sweden	27.10			Sweden	33.24	Sweden	33.79	Norway	19.67		
1973	Sweden	27.00			Sweden	33.24	Sweden	33.92	Norway	18.99		
1974	Sweden	26.83			Sweden	33.24	Sweden	33.72	Norway	18.95		
1975	Sweden	26.83	Sweden	26.92	Sweden	33.24	Japan	33.88	Norway	18.95	Norway	18.98
1976	Japan	26.15			Japan	32.91			Norway	18.82		
1977	Japan	25.18			Japan	31.83			Norway	18.28		
1978	Japan	24.39			Japan	31.06			Sweden	18.20		
1979	Japan	23.49			Japan	30.10			Japan	17.48		
1980	Japan	23.38			Japan	30.09			Japan	17.29		
1981	Japan	22.64			Japan	29.19			Japan	16.67		
1982	Japan	21.90			Japan	28.39			Japan	15.98		
1983	Japan	21.77			Japan	28.36			Japan	15.71		
1984	Japan	21.21			Japan	27.78			Japan	15.17		
1985	Japan	20.71			Japan	27.15			Japan	14.79		
1986	Japan	20.11			Japan	26.56			Japan	14.14		
1987	Japan	19.54			Japan	25.88			Japan	13.66		
1988	Japan	19.50			Japan	25.81			Japan	13.60		
1989	Japan	19.07			Japan	25.31			Japan	13.18		
1990	Japan	18.98			Japan	25.30			Japan	12.93		
1991	Japan	18.80			Japan	25.10			Japan	12.68		
1992	Japan	18.80	Japan	18.95	Japan	25.10	Japan	25.32	Japan	12.65		
1993	Japan	18.80	Japan	18.84	Japan	25.10	Japan	25.19	Japan	12.48		
1994	Japan	18.31			Japan	24.52			Japan	12.03		
1995	Japan	18.31	Japan	18.56	Japan	24.52	Sweden	24.67	Japan	12.03	Japan	12.28

© 2025 Karlsson O et al. *JAMA Health Forum*.

	Both sexes				Males				Females			
	Frontier (lowest ever)		Lowest PPD for year		Frontier (lowest ever)		Lowest PPD for year		Frontier (lowest ever)		Lowest PPD for year	
Year	Country	PPD, %	Country	PPD, %	Country	PPD, %	Country	PPD, %	Country	PPD, %	Country	PPD, %
1996	Japan	17.86			Japan	23.95			Japan	11.67		
1997	Japan	17.55			Sweden	23.52			Japan	11.47		
1998	Japan	17.55	Japan	17.63	Sweden	23.03			Japan	11.38		
1999	Japan	17.54			Sweden	22.36			Japan	11.32		
2000	Japan	16.90			Sweden	21.67			Japan	10.86		
2001	Japan	16.42			Sweden	21.52			Japan	10.56		
2002	Japan	15.98			Sweden	21.04			Japan	10.30		
2003	Japan	15.85			Sweden	20.61			Japan	10.11		
2004	Japan	15.53			Australia	20.02			Japan	10.01		
2005	Japan	15.47			Australia	19.61			Japan	9.95		
2006	Japan	15.01			Australia	19.00			Japan	9.68		
2007	Japan	14.77			Sweden	18.98			Japan	9.51		
2008	Japan	14.56			Australia	18.46			Japan	9.36		
2009	Japan	14.25			Sweden	18.00			Japan	9.09		
2010	Italy	13.97			Australia	17.61			Japan	9.09	Japan	9.14
2011	Italy	13.94			Sweden	17.39			Japan	9.09	Spain	9.27
2012	Japan	13.70			Sweden	16.82			Japan	8.87		
2013	Italy	13.35			Sweden	16.77			Japan	8.75		
2014	Italy	13.12			Sweden	16.41			South Korea	8.44		
2015	Japan	12.75			Norway	16.22			Japan	8.34		
2016	Japan	12.57			Sweden	16.09			South Korea	8.06		
2017	Japan	12.30			Norway	15.53			South Korea	7.73		
2018	Japan	12.18			Norway	15.26			South Korea	7.49		
2019	Japan	12.04			Sweden	14.82			South Korea	6.89		
2020	South Korea	11.76			Norway	14.59			South Korea	6.89	South Korea	7.04
2021	South Korea	11.43			Norway	14.34			South Korea	6.81		
2022	South Korea	11.43	Sweden	12.16	Norway	14.34	Sweden	14.53	South Korea	6.81	South Korea	8.07
2023	South Korea	10.22			Norway	13.12			South Korea	6.09		

Note: Probability of premature death (PPD) was defined as dying before age 70 years. The frontier is the lowest PPD ever observed up until and including each year (ie, increases across years were removed). The table also shows the lowest PPD and country with the lowest PPD within each year if different from the actual frontier. Countries with a population below 5 million in 2019 were not considered for being a frontier. United Arab Emirates, Hong Kong, and Switzerland were not considered for being a frontier since they have very large immigrant populations. Data source: UN WPP 2024 after 1950 and HMD 2024 before 1950.

© 2025 Karlsson O et al. *JAMA Health Forum*.

eTable 2. Regions

Central Asia	Central & Eastern Europe	Latin America & the Caribbean	Middle East & North Africa	North Atlantic	Sub-Saharan Africa	Western Pacific & Southeast Asia
Afghanistan	Albania	Anguilla	Algeria	Andorra	Angola	American Samoa
Azerbaijan	Armenia	Antigua and Barbuda	Bahrain	Austria	Benin	Australia
Kazakhstan	Belarus	Argentina	Egypt	Belgium	Botswana	Bangladesh
Kyrgyzstan	Bosnia and Herzegovina	Aruba	Iran	Bermuda	British Indian Ocean Territory	Bhutan
Mongolia	Bulgaria	Bahamas	Iraq	Canada	Burkina Faso	Brunei Darussalam
Pakistan	Croatia	Barbados	Israel	Channel Islands	Burundi	Cambodia
Tajikistan	Czechia	Belize	Jordan	Cyprus	Cabo Verde	China, Hong Kong SAR
Turkmenistan	Estonia	Bolivia	Kuwait	Denmark	Cameroon	China, Macao SAR
Uzbekistan	Georgia	Bonaire, Sint Eustatius and Saba	Lebanon	Faroe Islands	Central African Republic	China, Taiwan Province of China
	Hungary	Bouvet Island	Libya	Finland	Chad	Christmas Island
	Kosovo (under UNSC res. 1244)	Brazil	Morocco	France	Comoros	Cocos (Keeling) Islands
	Latvia	British Virgin Islands	Oman	Germany	Congo	Cook Islands
	Lithuania	Cayman Islands	Qatar	Gibraltar	Congo DR	Fiji
	Moldova	Chile	Saudi Arabia	Greece	Côte d'Ivoire	French Polynesia
	Montenegro	Colombia	State of Palestine	Greenland	Djibouti	Guam
	North Macedonia	Costa Rica	Syria	Guernsey	Equatorial Guinea	Heard Island and McDonald Islands
	Poland	Cuba	Tunisia	Holy See	Eritrea	Indonesia
	Romania	Curaçao	Türkiye	Iceland	Eswatini	Japan
	Russia	Dominica	United Arab Emirates	Ireland	Ethiopia	Kiribati
	Serbia	Dominican Republic	Yemen	Isle of Man	French Southern Territories	Lao
	Slovakia	Ecuador		Italy	Gabon	Malaysia
	Slovenia	El Salvador		Jersey	Gambia	Maldives
	Ukraine	Falkland Islands (Malvinas)		Liechtenstein	Ghana	Marshall Islands
		French Guiana		Luxembourg	Guinea	Micronesia
		Grenada		Malta	Guinea-Bissau	Myanmar
		Guadeloupe		Monaco	Kenya	Nauru
		Guatemala		Netherlands	Lesotho	Nepal
		Guyana		Norway	Liberia	New Caledonia
		Haiti		Portugal	Madagascar	New Zealand
		Honduras		Saint Pierre and Miquelon	Malawi	Niue
		Jamaica		San Marino	Mali	Norfolk Island
		Martinique		Spain	Mauritania	North Korea
		Mexico		Svalbard and Jan Mayen Islands	Mauritius	Northern Mariana Islands
		Montserrat		Sweden	Mayotte	Palau
		Nicaragua		Switzerland	Mozambique	Papua New Guinea
		Panama		United Kingdom	Namibia	Philippines
		Paraguay		Åland Islands	Niger	Pitcairn
		Peru			Nigeria	Samoa
		Puerto Rico			Rwanda	Singapore
		Saint Barthélemy			Réunion	Solomon Islands

© 2025 Karlsson O et al. *JAMA Health Forum*.

Central Asia	Central & Eastern Europe	Latin America & the Caribbean	Middle East & North Africa	North Atlantic	Sub-Saharan Africa	Western Pacific & Southeast Asia
		Saint Kitts and Nevis			Saint Helena	South Korea
		Saint Lucia			Sao Tome and Principe	Sri Lanka
		Saint Martin (French part)			Senegal	Thailand
		Saint Vincent and the Grenadines			Seychelles	Timor-Leste
		Sint Maarten (Dutch part)			Sierra Leone	Tokelau
		South Georgia and the South Sandwich Islands			Somalia	Tonga
		Suriname			South Africa	Tuvalu
		Trinidad and Tobago			South Sudan	US Minor Outlying Islands
		Turks and Caicos Islands			Sudan	Vanuatu
		United States Virgin Islands			Tanzania	Viet Nam
		Uruguay			Togo	Wallis and Futuna Islands
		Venezuela			Uganda	
					Western Sahara	
					Zambia	
					Zimbabwe	

© 2025 Karlsson O et al. *JAMA Health Forum*.

eAppendix 1. Economic Growth and Mortality

Economic growth and mortality decline are both part of a broader development process with many shared explanatory factors. Causal links between economic growth and health have also been suggested, running in both directions: Improved health can increase growth, since healthier populations are more productive and have greater incentives to both save and invest in their human capital (as they expect to live longer).[1,2] Economic growth can also improve health, for example, by improving living standards, allowing building and maintenance of important public health infrastructure, and increasing spending on medical treatments.[2] Therefore, we also assessed the PPD performance relative to the expected level of progress given economic development. We did this by integrating Preston curves—which show the cross-sectional relationship between PPD and aggregate income[3]—into our examination. Ultimately, health enhancing activities will be bounded by income, but countries at the same level of economic development can still achieve vastly different health outcomes due to contextual factors, such as prioritization of health spending, sub-national inequalities, and cultural and environmental factors.

© 2025 Karlsson O et al. *JAMA Health Forum*.

eAppendix 2. Estimating Preston Curves

Pooling all years and the 169 countries with available GDP data, we estimated a linear regression of PPD on (log) real per capita GDP and separate intercepts for each year. GDP data was missing for a few countries (16 countries 1970–1979, two countries 1980–1984, one country 1985–1992, and no country after 1992). Countries were equally weighted so results would not be overly influenced by large countries with distinct patterns, such as the United States and China. Technological advancements result in the intercepts shifting downward, such that in more recent years, lower PPD should be achieved for the same level of GDP. This shift in the Preston curve across time allows us to estimate how far behind or ahead regions were from the 2019 Preston curve given their GDP and PPD. (We observed a few cases where the intercepts moved slightly upward across years and adjusted these such that the intercept each year was the lowest ever observed since 1950.)

As a sensitivity analysis we allowed for a more flexible relationship between per capita GDP and PPD, where we regressed PPD on an intercept and log of per capita GDP for each year separately, obtaining year-specific intercepts and year-specific GDP slopes. Using this specification, the predicted PPD across the distribution of per capita GDP could intersect between different years, which goes against what would be suggested by the Preston curve (the same level of per capita GDP would generally not predict a lower PPD in an earlier year than a later year). Using this more flexible equation, we determined years behind or ahead for a given level of GDP the same as when using the Preston curves.

© 2025 Karlsson O et al. *JAMA Health Forum*.

eFigure 1. Lowest PPD, Frontier PPD, and Linear Prediction of Frontier PPD 1820-2019

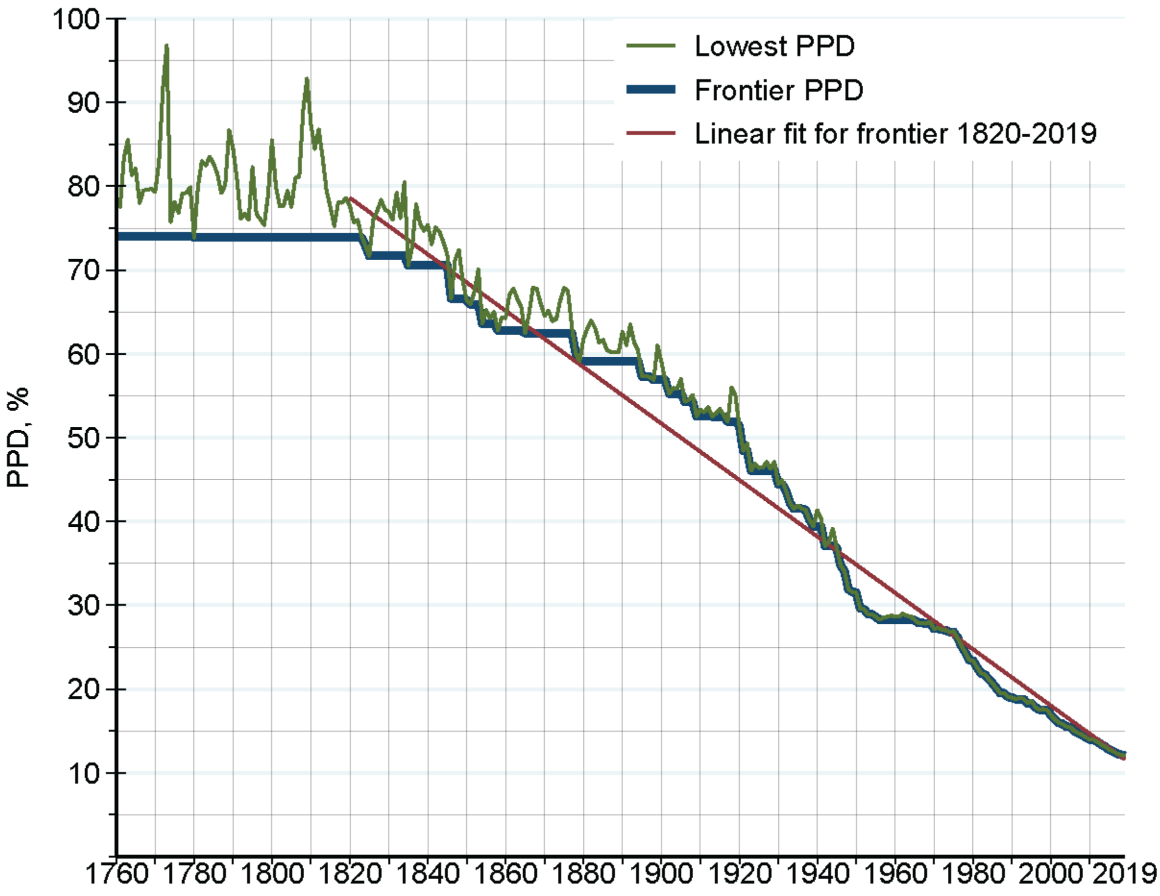

Note: Probability of premature death (PPD) was defined as dying before age 70 years. The frontier is the lowest PPD ever observed up until and including each year (ie, increases across years were removed). Countries with a population below 5 million in 2019 were not considered for being a frontier. Data source: UN WPP 2024 after 1950 and HMD 2024 before 1950.

© 2025 Karlsson O et al. *JAMA Health Forum*.

eFigure 2. Years Behind Frontier PPD in 2019 and 2023: Regions

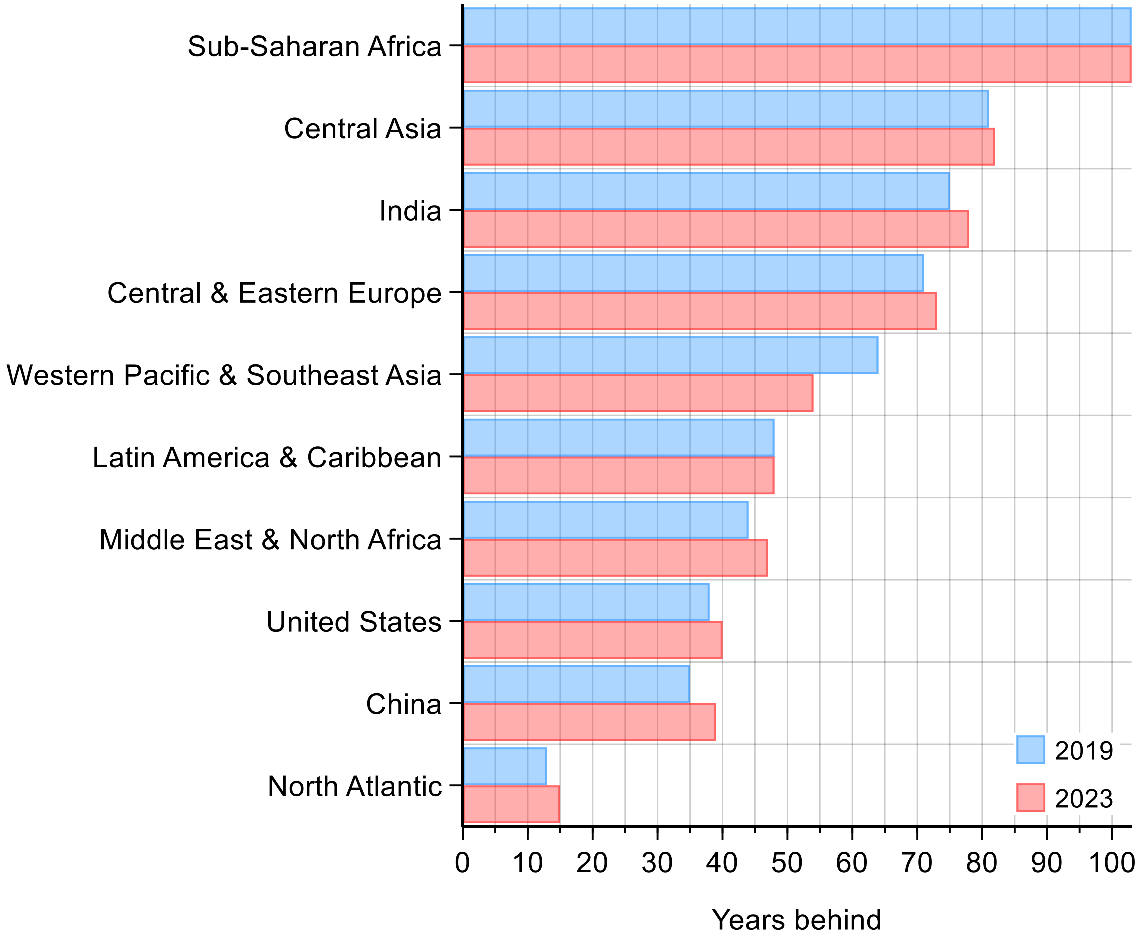

Note: Probability of premature death (PPD) was defined as dying before age 70 years. The frontier is the lowest PPD ever observed up until and including each year (ie, increases across years were removed). Countries with a population below 5 million in 2019 were not considered for being a frontier. Data source: UN WPP 2024 after 1950 and HMD 2024 before 1950.

© 2025 Karlsson O et al. *JAMA Health Forum*.

eFigure 3. Years Behind Frontier PPD in 2019 and 2023: 30 Most Populous Countries

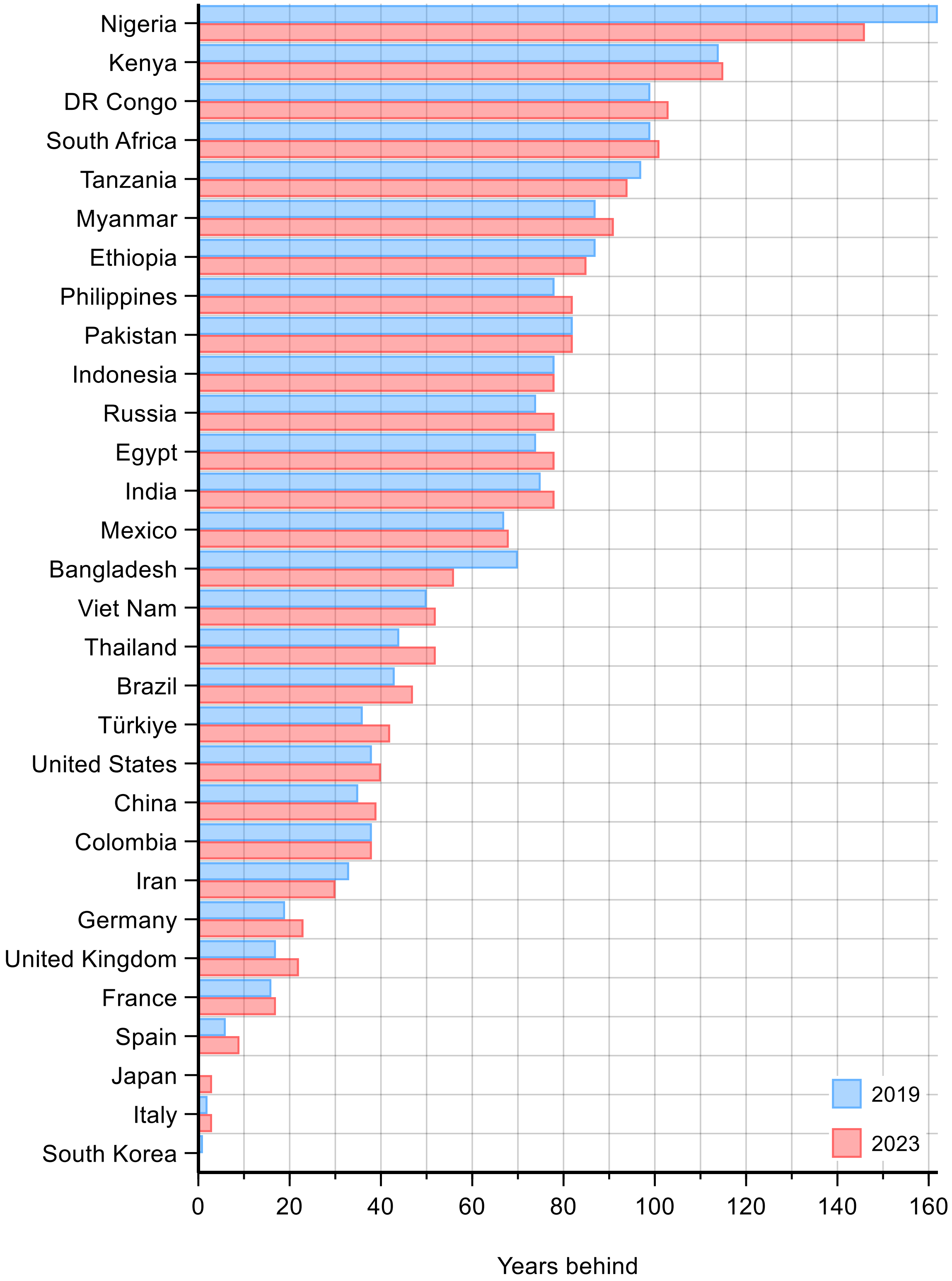

Note: Probability of premature death (PPD) was defined as dying before age 70 years. The frontier is the lowest PPD ever observed up until and including each year (ie, increases across years were removed). Countries with a population below 5 million in 2019 were not considered for being a frontier. Data source: UN WPP 2024 after 1950 and HMD 2024 before 1950.

© 2025 Karlsson O et al. *JAMA Health Forum*.

eFigure 4. Linear Frontier PPD Across Time (Line) and for Regions in 2019 (Markers)

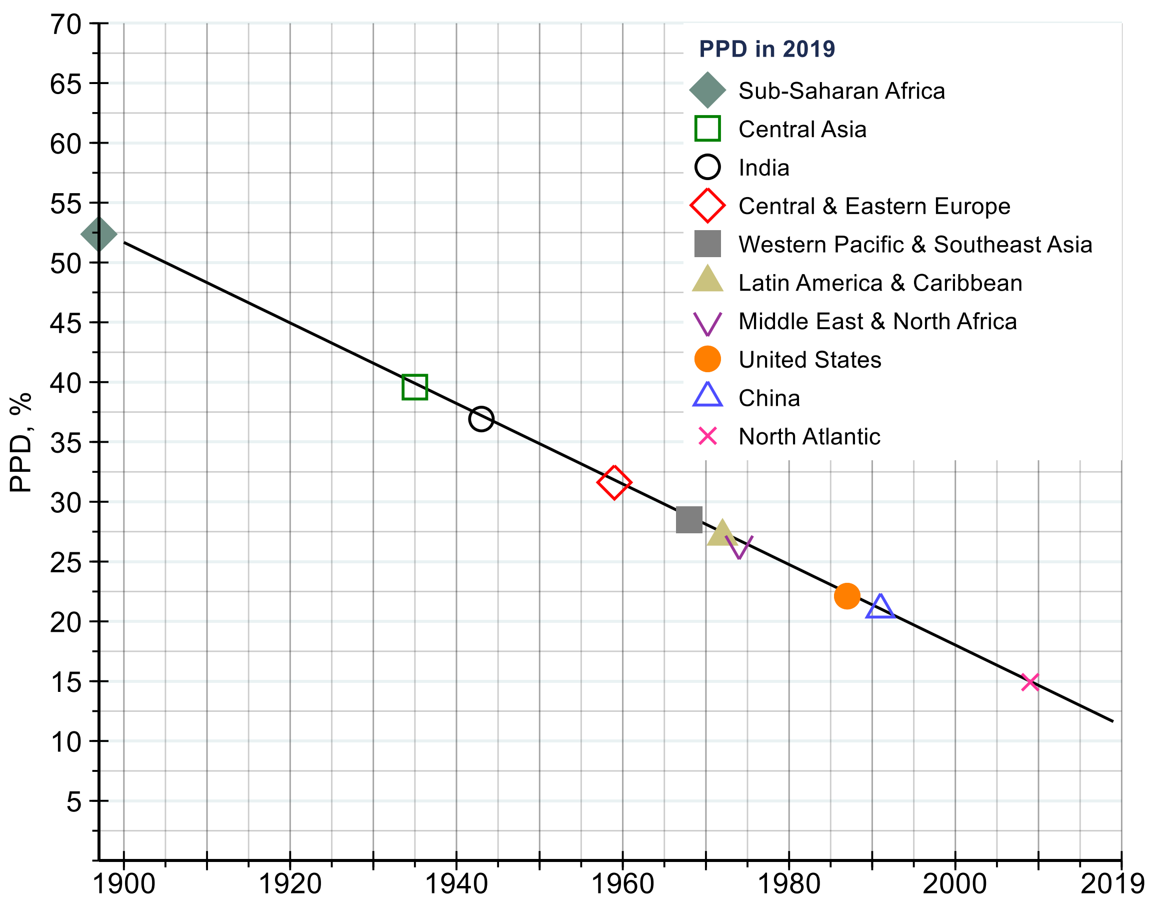

Note: Probability of premature death (PPD) was defined as dying before age 70 years. The line shows frontier PPD each year while the markers indicate PPD in 2019 for each location. The frontier is the lowest PPD ever observed up until and including each year (ie, increases across years were removed). Linear frontier is predicted from a regression of frontier PPD on year 1820–2019. Countries with a population below 5 million in 2019 were not considered for being a frontier. Data source: UN WPP 2024 after 1950 and HMD 2024 before 1950.

© 2025 Karlsson O et al. *JAMA Health Forum*.

eFigure 5. Years Behind Linear Frontier PPD 1970-2019

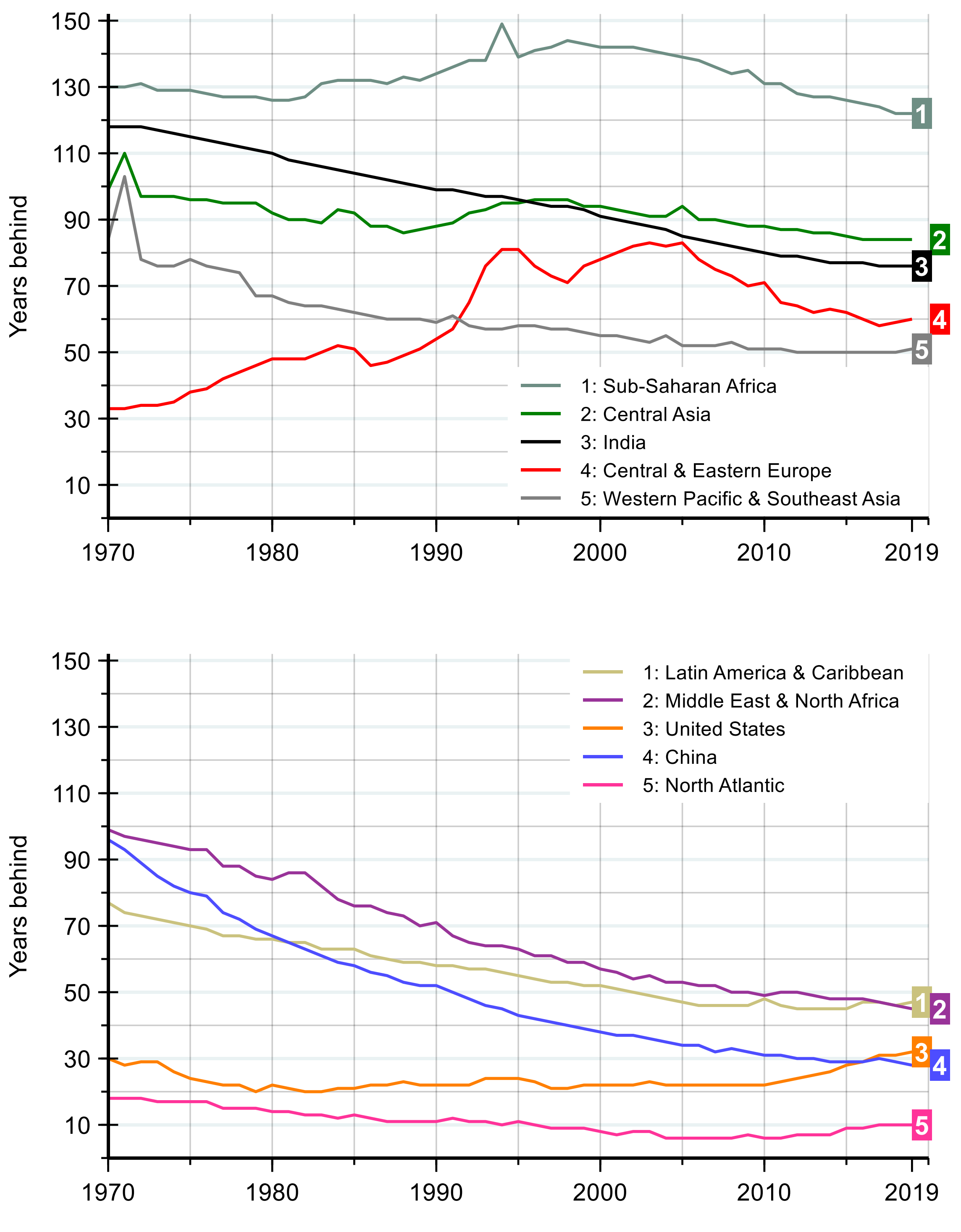

Note: Probability of premature death (PPD) was defined as dying before age 70 years. The frontier is the lowest PPD ever observed up until and including each year (ie, increases across years were removed). Linear frontier is predicted from a regression of frontier PPD on year 1820–2019. Countries with a population below 5 million in 2019 were not considered for being a frontier. Data source: UN WPP 2024 after 1950 and HMD 2024 before 1950.

© 2025 Karlsson O et al. *JAMA Health Forum*.

eFigure 6. Years Behind Linear Frontier PPD in 2000 and 2019: 30 Most Populous Countries

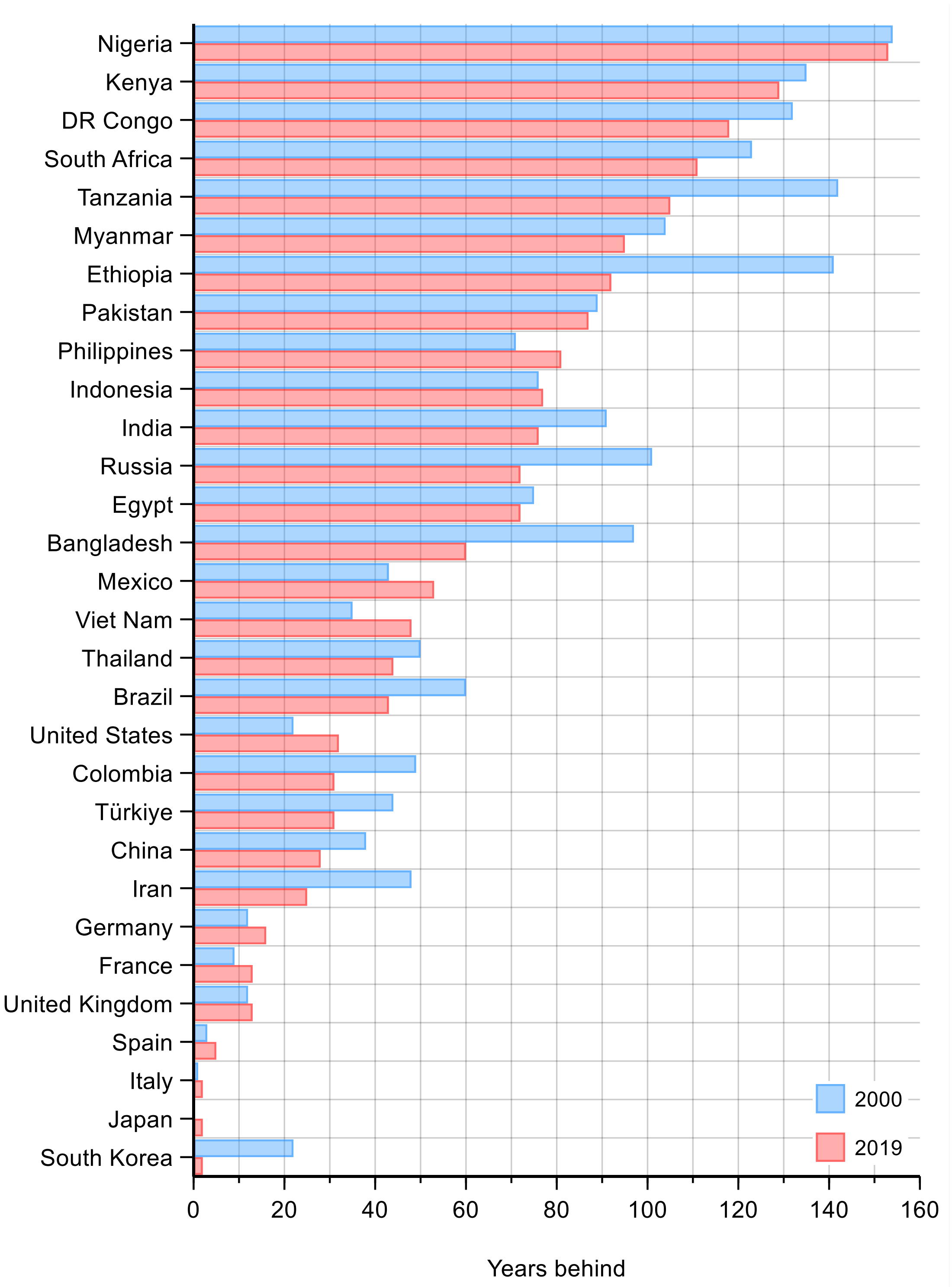

Note: Probability of premature death (PPD) was defined as dying before age 70 years. The frontier is the lowest PPD ever observed up until and including each year (ie, increases across years were removed). Linear frontier is predicted from a regression of frontier PPD on year 1820–2019. Countries with a population below 5 million in 2019 were not considered for being a frontier. Data source: UN WPP 2024 after 1950 and HMD 2024 before 1950.

© 2025 Karlsson O et al. *JAMA Health Forum*.

eFigure 7. Linear Frontier PPD Across Time (Line) and for Regions in 2019 (Markers): By Sex

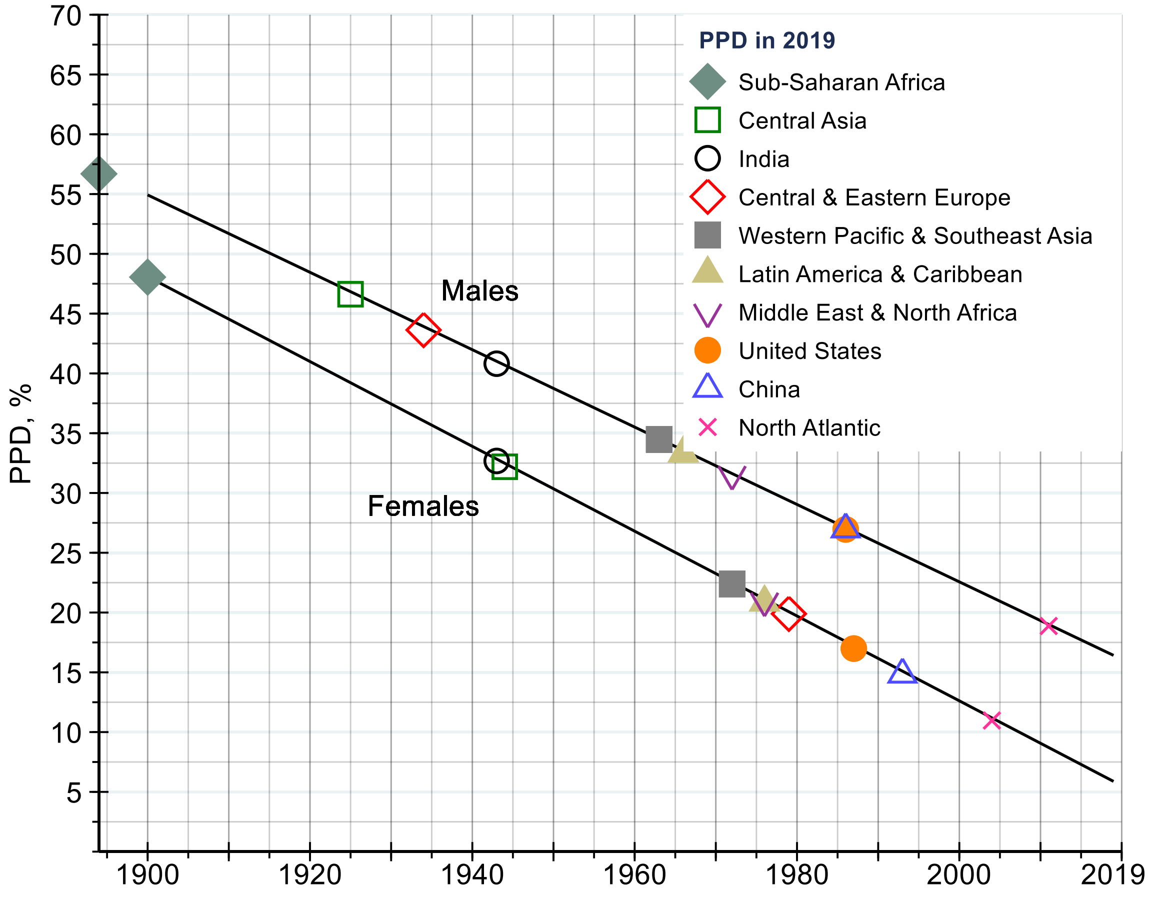

Note: Probability of premature death (PPD) was defined as dying before age 70 years. The line shows frontier PPD each year while the markers indicate PPD in 2019 for each location. The frontier is the lowest PPD ever observed up until and including each year (ie, increases across years were removed). Linear frontier is predicted from a regression of frontier PPD on year 1880–2019. Countries with a population below 5 million in 2019 were not considered for being a frontier. Data source: UN WPP 2024 after 1950 and HMD 2024 before 1950.

© 2025 Karlsson O et al. *JAMA Health Forum*.

eFigure 8. Years Behind or Ahead of PPD Expected for Level of Economic Development

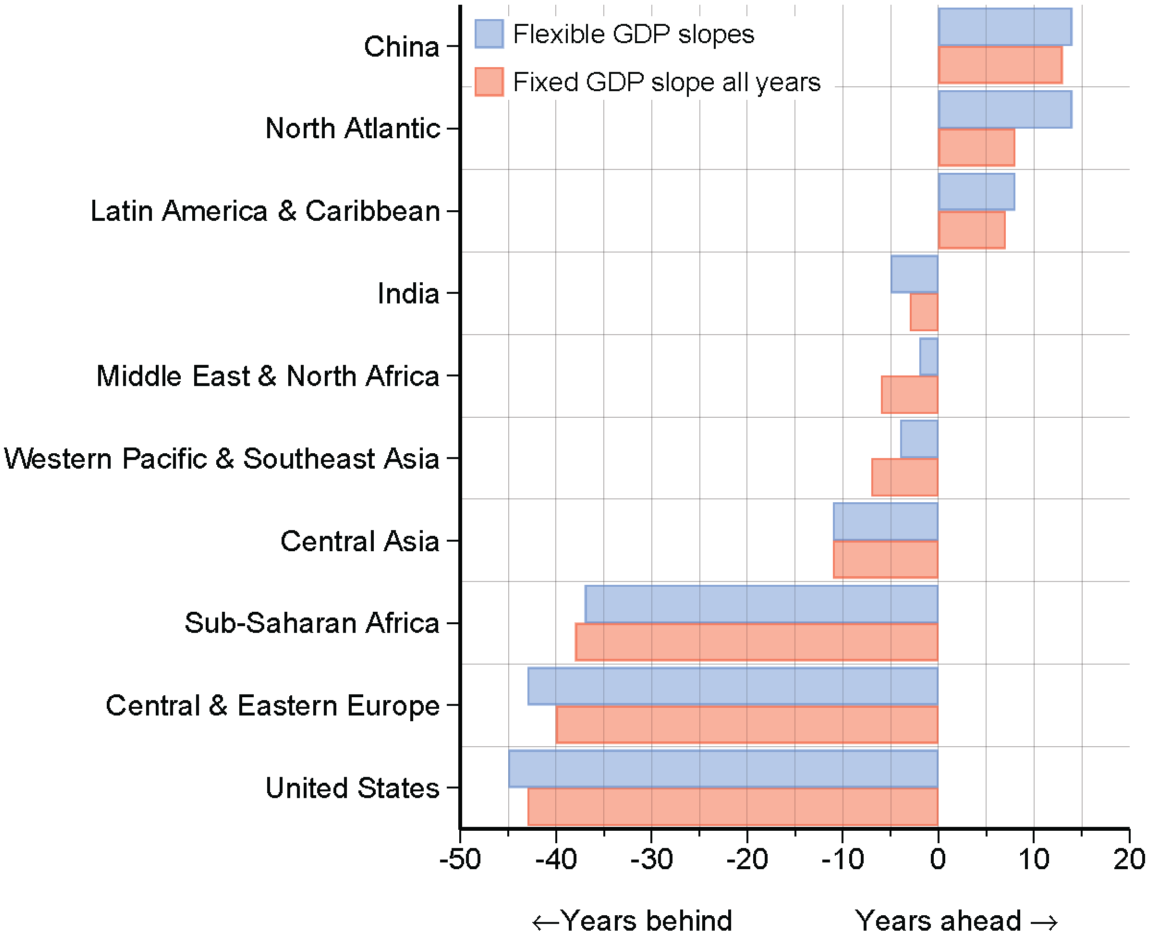

Note: Probability of premature death (PPD) was defined as dying before age 70 years. Preston curves were estimated for each year by regressing PPD on log of GDP (unweighted) with a separate intercept for each year (red bars). The slope for GDP is constant across years while the intercept varies across years. The intercepts were adjusted such that they never increased across years. The blue bars show predictions from more flexible regressions of PPD on GDP, where the GDP slopes could vary and intercepts could increase across years. Data source: Mortality data from UN WPP 2024; GDP data from the Maddison Project 2024.

eReferences

1. Bloom DE, Canning D. The Health and Wealth of Nations. *Science*. 2000;287(5456):1207-1209. doi:10.1126/science.287.5456.1207

2. Weil DN. Chapter 3 - Health and Economic Growth. In: Aghion P, Durlauf SN, eds. *Handbook of Economic Growth*. Vol 2. Handbook of Economic Growth. Elsevier; 2014:623-682. doi:10.1016/B978-0-444-53540-5.00003-3

3. Preston SH. The changing relation between mortality and level of economic development. *Popul Stud*. 1975;29(2):231-248.

© 2025 Karlsson O et al. *JAMA Health Forum*.
