# Supplementary material for: Global Disparities in Premature Mortality
Source: JAMA Health Forum. 2025 Oct 3;6(10):e253479. doi: 10.1001/jamahealthforum.2025.3479 (PMC12495499; doi:10.1001/jamahealthforum.2025.3479)
Supplement: Supplement 2. — Data Sharing Statement [file jamahealthforum-e253479-s002.pdf]

Data Sharing Statement

Karlsson. Global Disparities in Premature Mortality. *JAMA Health Forum*. Published October 03, 2025. doi:10.1001/jamahealthforum.2025.3479

Data
Data available: Yes
Data types: Data (not involving human participants)
How to access data: United Nations Population Prospects single age life tables are available at https://population.un.org/wpp/. Human Mortality Database is available at https://www.mortality.org/. The Maddison Project per capita GDP is available at https://www.rug.nl/ggdc/historicaldevelopment/.
When available: With publication

Supporting Documents
Document types: Statistical/analytic code
How to access documents: Codes used to produce the estimates in this paper is available at https://github.com/O-Karlsson/Global-Disparities-in-Premature-Mortality.
When available: With publication

Additional Information
Who can access the data: Anyone.
Types of analyses: All.
Mechanisms of data availability: The Human Mortality Database requires a simple registration. Without investigator support.
